# Comparative karyotypic analysis in the *Alstroemeria hookeri* Lodd. (Alstroemeriaceae) complex sensu Bayer (1987)

**DOI:** 10.1590/S1415-47572010005000012

**Published:** 2010-03-01

**Authors:** Carlos Baeza, Eduardo Ruiz, María Negritto

**Affiliations:** Departamento de Botánica, Facultad de Ciencias Naturales y Oceanográficas, Universidad de Concepción, ConcepciónChile

**Keywords:** *Alstroemeria hookeri*, complex, karyotype, polymorphisms, Chile

## Abstract

*Alstroemeria* L. (Alstroemeriaceae) is an American genus of monocots with two principal distribution centers in Chile and Brazil. In Chile, it is represented by about 32 species, most of them in central Chile, an area known for its high level of endemism. The “complex” *Alstroemeriahookeri* is endemic to Chile, where it is distributed from the Coquimbo to the Bío-Bío Region. We analyzed the karyotypes of 36 populations of this complex along its natural distribution. Ten metaphases per population were used for chromosome measurements. All analyzed subspecies presented a well defined asymmetric karyotype. The populations of *A. hookeri* subsp. *hookeri* collected in the coastal range of the Bío-Bío Region and the populations from the Central Valley of this Region (Pangal del Laja) presented striking morphological differences in the karyotype, mainly on chromosome 3. The population of *A. hookeri* subsp. *recumbens* from Pichicuy showed a polymorphism on chromosome 7, which differed from the other analyzed populations of this subspecies. Phenetic analysis suggested that *A. hookeri* subsp. *cummingiana*, which showed a more symmetrical karyotype and did not grow in sandy soil, should be alocated to *A. cummingiana* rather than considered as part of the *hookeri* complex.

## Introduction

*Alstroemeria* L. is a South American genus of Alstroemeriaceae that includes around 50 species found from Brazil to the Patagonian Region of Argentina and Chile in highly diverse habitats ranging from sea level to 4.000 m of altitude (Bayer, 1987; Ravenna, 1988; Sanso, 2002; Aagesen and Sanso, 2003). Central Chile is recognized as a center of diversity for this genus (Bayer, 1987), with satellite distributions occurring in central and eastern Brazil. Approximately 32 species grow in Chile; between 20° S and 53° S, with most of the taxa being found between 28° and 37° S (Bayer, 1987; Muñoz and Moreira, 2003). The great diversity of environments of this region has resulted in high levels of endemism in central Chile (Arroyo, 1995). *Alstroemeria* is one of the most diverse genera of Chiles vascular flora, providing an enormous and comprehensive floristic and morphological variability, especially in the coloration and ornamentation of the flowers, as evidenced by Muñoz and Moreira (2003).

The Chilean species of *Alstroemeria* have acquired economic relevance as ornamental plants, due to the beauty of their flowers (Buitendijk *et al.*, 1997). Many of the species have acquired considerable commercial value and are cultivated in different countries, such as Holland, Great Britain, Japan and the USA (Baeza *et al.*, 2007a). The main factors contributing to this success are the harvest durability of the flowers and the attractiveness of the perigonium.

Sanso (2002) published interesting data on the karyological analysis of seven Andean *Alstroemeria* taxa. In this study, supernumerary chromosomes (B chromosomes) were reported in most of the analyzed metaphases of *Alstroemeria hookeri* subsp. *recumbens* (Herbert) Bayer, but the other subspecies of the complex were not considered. B chromosomes had already been reported for *Alstroemeriaangustifolia* subsp. *angustifolia* (Buitendijk and Ramanna, 1996) and their occurrence may confer a selective advantage in relation to plants without them (Holmes and Bougourd, 1989, 1991)

The *A. hookeri* complex is endemic to Chile and occurs in the Central Chilean Zone, where the largest number of Chilean species are found and high levels of endemism exist (Villagrán *et al.*, 1994; Teneb *et al.*, 2004).

Four subspecies of *Alstroemeria hookeri* are recognized (Bayer, 1987): *A. hookeri* Lodd subsp. *hookeri* (from the Bío Bío and Maule Regions), *A. hookeri*. subsp. *recumbens* (Herbert) Bayer (endemic to the Valparaíso Region), *A. hookeri* subsp. *maculata* Bayer (endemic to the Coquimbo Region) and *A. hookeri* subsp. *cummingiana* (Herbert) Bayer from the Metropolitana, Valparaíso and Coquimbo Regions. Muñoz and Moreira (2003) recognized only three subspecies in the *A. hookeri* complex: *A. hookeri* subsp. *hookeri*, *A. hookeri* subsp. *maculata* and *A. hookeri* subsp. *recumbens*. *A*. *cummingiana* was maintained as a different species.

Baeza *et al.* (2007b) and Cajas *et al.* (2009) found differences between the karyotypes of populations of *A. hookeri* subsp. *hookeri* from the Central Valley and from the coastal range in the Bío-Bío Region of Chile. The karyotypes of the coastal and eastern populations were remarkably different, mainly regarding their chromosome 3. In the coastal range populations, this chromosome was telocentric, whereas in the eastern populations, it was metacentric. In addition, there were differences between chromosomes 4 and 8 of both karyotypes. Chromosome 4 was telocentric and chromosome 8 was submetacentric in the coastal range populations, whereas both chromosomes were subtelocentric in the eastern populations.

Given these previous data in the typical subspecies and the potential ornamental value of the *A. hookeri* complex, the objective of this work was to expand the comparative cytological analysis of all the subspecies of this complex (*sensu* Bayer, 1987).

## Material and Methods

###  Plant material

**Figure 1:**
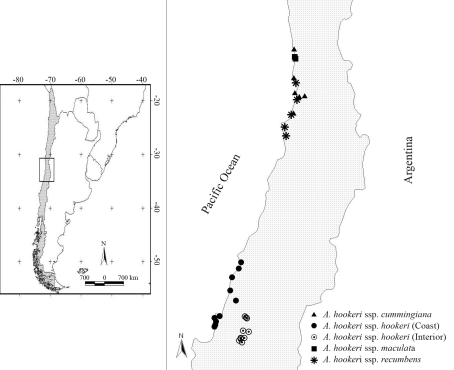
Geographic distribution of the 36 analyzed populations of the *Alstroemeriahookeri* complex.

Samples of 15 individuals from 36 populations of each subspecies were collected during November and December of 2007 and 2008 (Figure 1). The sources of the material studied and deposited in CONC (Herbarium code of the University of Concepción, Chile) are summarized in Supplementary Material.

### Methods

Root tips with one to two cm in length, obtained from plants cultivated in the greenhouse, were pre-treated with a solution of 8-hydroxiquinolein (2 mM) for 24 h at 4 °C. They were kept in ethanol/acetic acid (3:1) for 24 h and stored in 70% ethanol at -20 °C. After fixing, an acid hydrolysis was carried out with 0.5 N HCl for 22 min at 45 °C. The root tips were then washed three times with distilled water and were finally stained with 1% acetic orcein. Chromosome counting, analysis, and interpretation (ten metaphases per population) were carried under a Zeiss Axioskop microscope equipped with a digital video camera. The chromosomes were measured with the MicroMeasure 3.3 software (Reeves, 2001) and were classified according to their relative arm lengths (Levan *et al.*, 1964). The TCL (total chromosomes length plus the standard deviation in μm) was obtained for each population; the AsK % (asymmetry index defined by Arano and Saito, 1980), TF% (asymmetry index defined by Huziwar, 1962), and Syi (asymmetry index defined by Venora *et al.*, 2002) were calculated.

Total chromosome length (TCL) was calculated as the percentage of the total genomic length corresponding to a haploid set. Photographs were processed with the Paint Shop Pro X2 software. The software NTSyS-pc (Numerical Taxonomic System of Multivariate Statistical Programs; Rohlf (2005) was used to perform a phenetic cluster analysis by UPGMA.

## Results and Discussion

**Figure 2:**
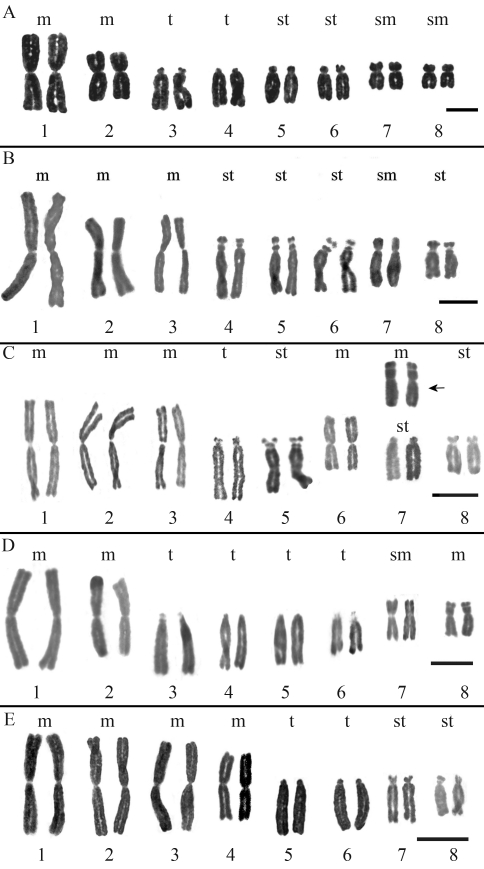
Karyotypes of the *Alstroemeria hookeri* complex populations: (a) *A. hookeri* subsp. *hookeri* from the coastal area of the Bío-Bío and Maule Regions of Chile (populations 4181, 4182, 4202, 4211, 4220, 4221, 4222, 4224, 4226, 4227, 4235, 4286 and 4287); (b) *A.hookeri* subsp. *hookeri* from the Central Valley of the Bío-Bío Region of Chile (populations 4175, 4187, 4189, 4212, 4214, 4215, 4216, 4217, 4218 and 4219); (c) *A.hookeri* subsp. *recumbens* (populations 4271, 4273, 4275, 4283 and 4284); (d) *A.hookeri* subsp. *maculata* (populations 4277 and 4278) and (e) *A.* *hookeri* subsp. *cummingiana* (populations 4272, 4274, 4276, 4279, 4281 and 4282). Bar = 10 μm.

**Figure 3:**
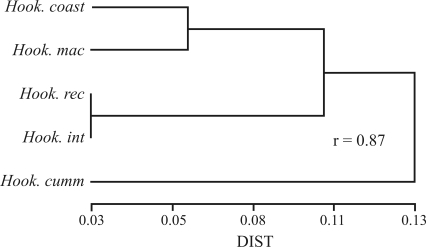
Phenogram obtained with NTSys-PC, with UPGMA and DIST coefficient.

All the analyzed samples of 36 populations had 2n = 2x = 16 chromosomes. Each subspecies in the complex had a different karyotype, which reflected in different asymmetry indexes and different total chromosome length (TCL) values (see Figure 2 and Table 1). Intra-subspecific variation was detected in *Alstroemeriahookeri* subsp. *recumbens* and *A.hookeri* subsp. *hookeri*. The former had four metacentric, two subtelocentric, one satellited subtelocentric and one telocentric chromosome pairs (4m + 2st + 1st-sat + 1t), but samples from the population of Pichicuy (4275) showed a different morphology in chromosome 7 (Figure 2c). More consistent differences were found in *A.hookeri* subsp. *hookeri.* All the populations of this taxon from the coastal range of the Bío-Bío Region had a karyotype with 2m + 2sm + 2st + 2t chromosome pairs (Figure 2a) and all the populations from the Central Valley had 3m + 1sm + 4st-sat chromosome pairs (and a more symmetrical karyotype; Figure 2b, Table 1). Although the most conspicuous difference between coastal and Central Valley populations was on chromosome 3, small but consistent differences in chromosomes 4 and 8 were also found. No intra-subspecific differences were detected in the other two subspecies. *A. hookeri* subsp. *maculata* had an asymmetric karyotype with a 2m + 1m-sat + 1sm + 1st-sat + 2t + 1t-sat chromosome set (see Table 1 and Figure 2d). *A*. *hookeri* subsp. *cummingiana* had the most symmetric karyotype with 4m + 1st + 1st-sat + 2t chromosome pairs (Figure 2e) and a higher TCL value (Table 1). The UPGMA phenogram (Figure 3) shows the phenetic relationships among populations based on karyotypic data. The closest relationships among populations were those between *A. hookeri* subsp. *hookeri* from the Central Valley of the Bío-Bío Region and *A. hookeri* subsp. *recumbens*, and between *A. hookeri* subsp. *hookeri* from the coastal range and *A. hookeri* subsp. *maculata*. The subspecies with the most divergent karyotype was *A*. *hookeri* subsp. *cummingiana,* as shown by its largest phenetic distance from the complex.

The *Alstroemeria hookeri* complex is a group of morphologically very similar subspecies, which typically grow in sandy soils, mostly near the coastal zone. According to Bayer (1987), the *Alstroemeria hookeri* complex consists of *A. hookeri* subsp. *hookeri*, *A. hookeri* subsp. *recumbens*, *A. hookeri* subsp. *maculata* and *A. hookeri* subsp. *cummingiana*. Among these taxa, *A. hookeri* subsp. *cummingiana* is the only one which does not grow in sandy soils, but rather in a brownish-grey non-calcium soil, with a lightly acidic, pink to light brownish-red A horizon ,and a light brownish-red or dirty red B horizon (Soil Survey Staff, 1999). During the development of this research, three populations from the Region of Valparaíso (Region V of Chile) were initially mistaken for *A. hookeri* subsp. *recumbens*, but later recognized as a new species of *Alstroemeria* from Chile, *Alstroemeria novoana* (Negritto M, Baeza C, Ruiz E and Novoa P, unpublished data). The new species also grows in soils similar to those where *A. hookeri* subsp. *cummingiana* is found and never in sandy soils. Many samples (ten populations) were collected in the sector Pangal del Laja, located in the central depression of the Bío-Bío Region (Supplementary Material). These populations have been considered *A. hookeri* subsp. *hookeri* for a long time, but, when thoroughly tested by combined cytological (Cajas *et al.*., 2009), morphological and isoenzymatic analyses (Ruiz E, Balboa K, Negritto M, Briceño V and Baeza M, unpublished data), these populations revealed enough features to allow their classification as a new subspecies within the *A. hookeri* complex. This new subspecies also grows in sandy soils and corresponds to populations 4175, 4187, 4189, 4212, 4214, 4215, 4216, 4217, 4218 and 4219 (Table 1). The ability to grow in such soils is therefore an exclusive feature of plants from the *A. hookeri* complex.

Previous cytogenetic studies in this complex have reported the presence of B chromosomes in *A. hookeri* subsp. *recumbens* (Sanso, 2002). Such chromosomes have been reported in other complexes such as *A. angustifolia* subsp. *angustifolia* (Buitendijk and Ramanna, 1996), a common species in the Region of Valparaíso, very similar to *A. hookeri* subsp. *recumbens*. After analyzing about 1000 metaphases in all subspecies of the complex, we found no evidence of B chromosomes in *Alstroemeria hookeri*. Sanso (2002) indicated that the material was collected in Longotoma, Region of Valparaíso, at 225 m, an unlikely habitat for *A. hookeri* subsp. *recumbens* because this plant grows in sandy soil and very close to the coast. Therefore, the presence of B chromosomes must be considered with caution in the *A. hookeri* complex. Many of the publications on the cytology of *Alstroemeria* from Chile have used plants grown in European greenhouses, sometimes with identification errors leading to mistakes like the one mentioned above. More work should be carried out on wild populations of *Alstroemeria* because cultivated material may present remarkable changes in leaf morphology and tepal color, which may lead to species misidentification.

The phenogram in Figure 3 shows three groups of populations. The first group is formed by *A. hookeri* subsp. *hookeri* from the coast of the Bío-Bío Region and by *A. hookeri* subsp. *maculata* from the Coquimbo Region. Both subspecies showed very similar karyotypes; which had identical pairs 1 through 4, pairs 5, 6 and 8 differing in their morphology (Figure 2a and 2d) and.a submetacentric chromosome 7. The total chromosome length (μm) was a distinguishing character because its value was much higher in *A. hookeri* subsp. *hookeri* than in *A. hookeri* subsp. *maculata* (Table 1). Both subpecies grow in coastal areas very close to the sea. The second group is composed by *A. hookeri* subsp. *recumbens* from the Valparaiso Region and by *A. hookeri* subsp. *hookeri* from the Central Valley of the Bío-Bío Region (the new subspecies within the complex). These two taxa were the most phenetically similar within the complex. Their first three chromosome pairs were identical, all metacentric and the largest in the karyotype, while pairs 4, 6 and 7 showed differences in morphology. A remarkable difference was observed on chromosome 6: it was a metacentric in *A. hookeri* subsp. *recumbens* from the Central Valley of the Bío-Bío Region, a unique feature in the complex (Figure 2c), whereas it was a subtelocentric with microsatellites on the short arms in *A. hookeri* subsp. *hookeri* (Figure 2b). Chromosomes 5 and 8 had the same morphology (st) in both taxa. Quantitatively, the TCL was much higher in *A. hookeri* subsp. *hookeri*. Population 4275 of *A. hookeri* subsp. *recumbens*, collected in Pichicuy, Petorca Province (northernmost distribution area of the subspecies), presented a marked polymorphism on chromosome 7, which was a metacentric with microsatellites on the short arms (Figure 2c). This chromosome was very stable in this population and did not appear in any other populations of this taxon throughout its distribution range, which showed a subtelocentric 7. Polymorphisms among homologous chromosomes has been detected in other species, such as: *Placea amoena* (Baeza and Schrader, 2004), *Brachycome* (Houben *et al.*, 2000), *Alstroemeria* (Buitendijk *et al.*, 1998) and *Scilla* (Greilhuber and Speta, 1976), among others. However, the presence of different homologous chromosomes in different populations of the same taxon is not frequent and has only been detected in the populations of *A. hookeri* subsp. *hookeri* from the coast and from the Central Valley of the Bío-Bío Region (Cajas *et al.*, 2009). Evolutionary divergence is likely to be occurring in this population of Pichicuy, which already presents chromosome variation, but no phenotypic differences yet, These results support comparative population studies of naturally growing plants throughout the distribution range of a taxa, such as *A. hookeri* subsp. *hookeri*.

*Alstroemeria hookeri* subsp. *cummingiana* appeared as the most distantly related taxon in relation to the other members of the *A. hookeri* complex. It is the only taxon with metacentric chromosomes 1-4 (Figure 2e) and it had the smallest ASK% value (64.2), meaning that it has the most symmetrical karyotype in the group. This cytogenetic feature, combined with the habitat and floral morphology of *A. hookeri* subsp. *cummingiana*, allowed us to conclude that this taxon should not be part of the *A. hookeri* complex, but rather classified as *Alstroemeria cummingiana* Herbert, as previously noted by Muñoz and Moreira (2003).

## Supplementary Material

The following online material is available for this article:

List of sampling sites

This material is made available as part of the online article from http://www.scielo.br.gmb.

## Figures and Tables

**Table 1:** Karyotype features of the subspecies of *Alstroemeria hookeri*.

Karyotype characteristics	*A. hookeri* subsp*. hookeri* (Coast)	*A. hookeri* subsp*. hookeri* (Central Valley)	*A. hookeri* subsp*. recumbens*	*A. hookeri* subsp*. maculata*	*A. hookeri* subsp*. cummingiana*
Karyotype formula	2m + 2sm + 2st + 2t	3m + 1sm + 4st-sat	4m + 2st + 1st-sat + 1t	2m + 1m-sat + 1sm + 1st-sat + 2t + 1t-sat	4m + 1st + 1st-sat + 2t
TCL (μm)	180.82 ± 8.2	251.16 ± 7.6	179.36 ± 7.2	157.06 ± 5.8	184.12 ± 5.5
AsK %	71.50	67.02	65.90	71.30	64.20
TF%	28.50	32.98	34.10	28.70	35.80
Syi%	39.84	49.21	51.65	40.28	55.74

TCL (μm) = Total chromosome length plus standard deviation. AsK% = Asymmetry index of Arano and Saito (1980), TF% = Asymmetry index of Huziwara (1962), Syi = Asymmetry index of Venora *et al.* (2002).
