# Supplementary material for: Comparative karyotypic analysis in the Alstroemeria hookeri Lodd. (Alstroemeriaceae) complex sensu Bayer (1987)
Source: Genet Mol Biol. 2010 Mar 1;33(1):119–24. doi: 10.1590/S1415-47572010005000012 (PMC3036094; doi:10.1590/S1415-47572010005000012)
Supplement: Supplementary file 1 — List of sampling sites [file gmb-33-1-119-suppl1.pdf]

Annex 1

Populations of Alstroemeria hookeri subsp. hookeri from de coastal range of the Regions of Bío Bío and Maule

VII Región. Provincia de Cauquenes. Tregualemu. 20 m, (36°00' S-72°46' W). C. Baeza 4285. Curanipe. 23 m, (35°50' S-72°37' W). C. Baeza 4286. Chanco. Reserva Forestal Francisco Albert. Playa Monolito. 2 m, (35°43' S-72°34' W). C. Baeza 4287. VIII Región. Provincia de Concepción. Lenga, 5 m, (36°46' S-73°09' W). C. Baeza 4181. Camino de San Pedro a Coronel, sector Stadio Italiano, 30 m, (36°54' S-73°08' W). C. Baeza 4182. Camino de San Pedro a Coronel, sector cruce hacia el peaje, 30 m, (36°57' S-73°09' W). C. Baeza 4202. Camino de San Pedro a Coronel, sector Bomba de Bencina YPF, 30 m, (36°56' S-73°09' W). C. Baeza 4211. Costanera, entre Las Areneras y la entrada a Petrox (Hualpén). Bosquete de Boldo, Litre y Pelú. 10 m, (36°47' S-73°06' W). C. Baeza 4220. Hualpén. Parque. Recinto Universitario, 6 m, (36°47' S-73°10' W). 18-1-2005. Baeza 4235. Pedro de La Paz, frente a la entrada a Boca Sur. 5 m, (36°50' S-73°07' W). C. Baeza 4221. Talcahuano, Isla Rocuant, 2 m, (36°44' S-73°02' S). C. Baeza 4222. Provincia de Ñuble. Colmuyao, playa 2m, (36°44' S-73°02' W). C. Baeza 4224. Cobquecura, frente a la Lobería 2m, (36°15' S-72°48' W). C. Baeza 4226. Trehuaco, salida sur, 6 m, (36°26' S-72°40' W). C. Baeza 4227.

Populations of Alstroemeria hookeri subsp. hookeri from the Central Valley in the Region of Bío Bío

VIII Región. Provincia de Biobío. Comuna de Quillón, Puente El Roble, 64 m, (36°45' S-72°25' W). C. Baeza 4175. Yumbel. Camino Estación Yumbel hacia Puente Perales, 102 m, (37°09' S-72°32' W). C. Baeza 4187. Camino Estación Yumbel hacia Puente Perales, 120 m, (37°11' S-72°34' W). C. Baeza 4189. Camino de Yumbel hacia la carretera 5 Sur, 100 m, (37°08' S-72°27' W). C. Baeza 4212. Camino de Laja hacia la carretera 5 Sur, 100 m, (37°13' S-72°30' W). C. Baeza 4214. Yumbel, salida sur, 100 m, (37°08' S-72°32' W). C. Baeza 4215. Camino entre Yumbel y Cabrero, 2 km al Este de Puente Ibáñez, 100 m, (37°00' S-72°29' W). C. Baeza 4216. Camino de Cabrero a Bulnes, 3,5 km desde Cabrero, orilla ruta 5 sur 100 m, (37°01' S-72°21' W). C. Baeza 4217. Camino hacia Quillón, sector Los Alemanes, 100 m, (36°46' S-72°24' W). C. Baeza 4218. Entrada a Quillón, casi al frente de la Laguna Avendaño, 70 m, (36°44' S-72°27' W). C. Baeza 4219.

Populations of Alstroemeria hookeri subsp. recumbens

V Región. Provincia de Valparaíso. Dunas de Concón. Santuario. 103 m, (32°56'/71°32'). C. Baeza 4271. Playa Quintay. 6 m, (33°10'/71°40'). C. Baeza 4284. Quintero. Sector Maitencillo. 74 m, (32°39'/71°25'). C. Baeza 4273. Provincia de Petorca. Pichicuy. 10 m, (32°20'/71°27'). C. Baeza 4275. Provincia de San Antonio. Algarrobo. Playa Mirasol. 2 m. (33°20'/71°38'). C. Baeza 4283.

Populations of Alstroemeria hookeri subsp. cummingiana

IV Región. Provincia de Choapa. Km 249,5 al norte de Los Vilos. 140 m, (31°42'/71°31'). C. Baeza 4279. V Región. Provincia de Valparaíso. Entre Mantagua y el puente del rio Aconcagua, orillas del camino. 45 m, (32°54'/71°29'). C. Baeza 4272. Provincia de Petorca. Zapallar, frente al cementerio. 38 m, (32°31'/71°28'). C. Baeza 4274. Los Molles, calle La Estrella Nº 743. 40 m, (32°14'/71°30'). C. Baeza 4276. Camino Maitencillo, Catapilco, km 10,86. 56 m, (32°35'/71°22'). C. Baeza 4281. Inicio Cuesta El Melón, km 137, bajada norte. 169 m, (32°34'/71°15'). C. Baeza 4282.

Populations of Alstroemeria hookeri subsp. maculata

IV Región. Provincia de Choapa. Los Vilos. Playa Matagorda, al final, bajo el ducto de cobre de Minera Pelambre. 2 m, (31°53'/71°29'). C. Baeza 4277. Extremo norte playa Agua Amarilla, Mal Paso, en terraza oceánica. 50 m, (31°51'/71°30'). C.Baeza 4278.
